# Characterization of Serum Metabolome and Proteome Profiles Identifies SNX5 Specific for Pregnancy Failure in Holstein Heifers

**DOI:** 10.3390/life12020309

**Published:** 2022-02-18

**Authors:** Kazuya Kusama, Rulan Bai, Yuta Matsuno, Atsushi Ideta, Toshihiro Sakurai, Kentaro Nagaoka, Masatoshi Hori, Kazuhiko Imakawa

**Affiliations:** 1 Department of Endocrine Pharmacology, Tokyo University of Pharmacy and Life Sciences, Tokyo 192-0392, Japan; kusamak@toyaku.ac.jp; 2 College of Veterinary Medicine, China Agricultural University, Beijing 100193, China; rulan@cau.edu.cn; 3 Research Institute of Agriculture, Tokai University, Kumamoto 862-8652, Japan; yuta.matsuno@tsc.u-tokai.ac.jp; 4 Zen-Noh Embryo Transfer Center, Fukuoka 810-0001, Japan; ideta-atsushi@zennoh.or.jp; 5 School of Pharmaceutical Science, Ohu University, Fukushima 963-8611, Japan; t-sakurai@pha.ohu-u.ac.jp; 6 Laboratory of Veterinary Physiology, Cooperative Department of Veterinary Medicine, Faculty of Agriculture, Tokyo University of Agriculture and Technology, Tokyo 183-8509, Japan; nagaokak@cc.tuat.ac.jp; 7 Laboratory of Veterinary Pharmacology, Graduate School of Agricultural and Life Sciences, The University of Tokyo, Tokyo 113-8657, Japan; horimasa@g.ecc.u-tokyo.ac.jp

**Keywords:** sorting nexin 5, pregnancy loss, Holstein heifer, metabolome analysis, proteome analysis

## Abstract

Pregnancy loss predominantly occurs during the first 3–4 weeks due to fertilization failure or early embryonic losses in cattle. Insufficient biochemical communication between conceptus (embryo plus extraembryonic membranes) and endometrium has been suspected as the primary cause for early embryonic losses. If molecules regulating this communication were identified, molecular mechanisms associated with early pregnancy losses could be better understood. To identify candidate molecules as detection markers of non-pregnant or females undergoing embryonic loss, peripheral blood from embryo-transferred heifers on day 7 (day 0 = day of estrus) were collected on days 17 (pre-attachment), 20 (during attachment), and 22 (post-attachment), which were subjected to metabolome and global proteome iTRAQ analyses. The metabolome analysis partly divided serum components into pregnant or not. In the iTRAQ analysis, heatmap analysis with top 25 proteins was separated into pregnant or not on day 20 or 22. Furthermore, receiver operating characteristic curve (ROC) analysis identified five candidate proteins detecting non-pregnant heifers, of which SNX5 in day 22 serum had the highest area under the curve (AUC): 0.983. We also detected SNX5 in day 22 serum from non-pregnant heifers using western blotting. These results suggest that high SNX5 in day 22 serum could predict early pregnancy loss in heifers.

## 1. Introduction

In cattle, pregnancy loss predominantly occurs during the first three to four weeks of gestation due to fertilization failure or early embryonic losses during the period of luteolysis or maternal recognition of pregnancy that occurs from day 14 to day 24 (day 0 = day of estrus) [1]. The latter is considered insufficient biochemical communication between conceptus (embryo plus extraembryonic membranes) and endometrium. Regardless of the use of methods such as artificial insemination (AI) and in vivo- or in vitro-fertilization, followed by embryo transfer (ET), pregnancy rates remain approximately 50%, and thus, half of the remaining cattle experiences corpus luteum (CL) demise. In ruminant ungulates, interferon-tau (IFNT) contributes to the prevention of luteolysis by attenuating pulsatile secretion of endometrial prostaglandin F2α (PGF) [1] through the downregulation of estrogen receptor and the subsequent estrogen-induced oxytocin receptor expression [2,3], resulting in continued secretion of progesterone (P4) from the CL and pregnancy establishment [4].

Data has been accumulated that although IFNT is generally considered not escaping from the uterine lumen, IFNT has recently been found to upregulate the expression of interferon stimulated genes (ISGs) in the endometrium [5], luteal cells [6], liver [7] as well as peripheral blood mononuclear cells (PBMCs) and polymorphonuclear cells (PMNs) [8]. Expression of ISG transcripts, including *ISG15*, *OAS1*, *MX1* and *MX2*, has been found to increase on day 15, peaks on day 20 and declines on day 22, of which expression is closely associated with those of IFNT [9,10,11].

Based on these results, intensive research has been conducted on the development of pregnancy diagnosis methods in AI-cattle. These include CL examination by Doppler ultrasonography [10], ISG expression in PBMCs and PMNs [8], and microRNA in extracellular vesicles [12]. All of these researches have provided sufficient results to differentiate pregnant animals from non-pregnant (NP) ones as early as day 20 post AI. However, these methods require equipment, or isolation of immune cells and extracellular vesicles, from which candidate transcripts must be evaluated.

Development of a method effectively identifying pregnant cattle from NP ones, which go through AI or ET procedure, requires, (a) the method to be convenient, and to be able to be used in the field, and (b) the method to be able to identify pregnant or NP cattle before or on day 22, the length of regular estrous cycles. In this regard, pregnancy associated glycoproteins (PAGs) would be ideal to identify pregnant from NP ones. However, the pregnancy diagnosis with the PAGs method can be conducted on or after day 28. It would thus be desirable if serum components or secreted body fluids, which could be used to develop a method for pregnancy diagnosis, are identified.

To develop a simple method for pregnancy diagnosis, which detects pregnancy status on days 20–22 and could be used in the field, we opted to study serum components in pregnant and NP heifers. During the period of maternal recognition of pregnancy, conceptuses go through elongation, attachment, and implantation to the maternal endometrium [13]. These changes in conceptuses of the ruminants in utero could reflect some changes in metabolites and/or proteins in the peripheral blood, if highly sensitive methods were applied to detect such changes. We therefore hypothesized that non-pregnant heifers without conceptuses could be identified through changes in the peripheral blood components. To test this hypothesis, blood samples were taken from Holstein heifers, which went through ET procedure with in vivo-fertilized trophoblast on day 7, and later the status of pregnancy or NP was evaluated. This study particularly examined conceptus survival or loss during the period corresponding to the maternal recognition of pregnancy.

## 2. Results

### 2.1. Metabolic Analysis of Bovine Peripheral Blood during the Peri-Implantation Period

A total of 20 heifers, which had regular estrous cycles and responded to the PGF2α treatment, were subjected to this study, from which 17 heifers went through the ET procedure while the remaining three did not. Among 17 ET heifers, 12 heifers became pregnant and five were found as NP heifers, 70% pregnancy rate (Figure S1).

**Figure 1:**
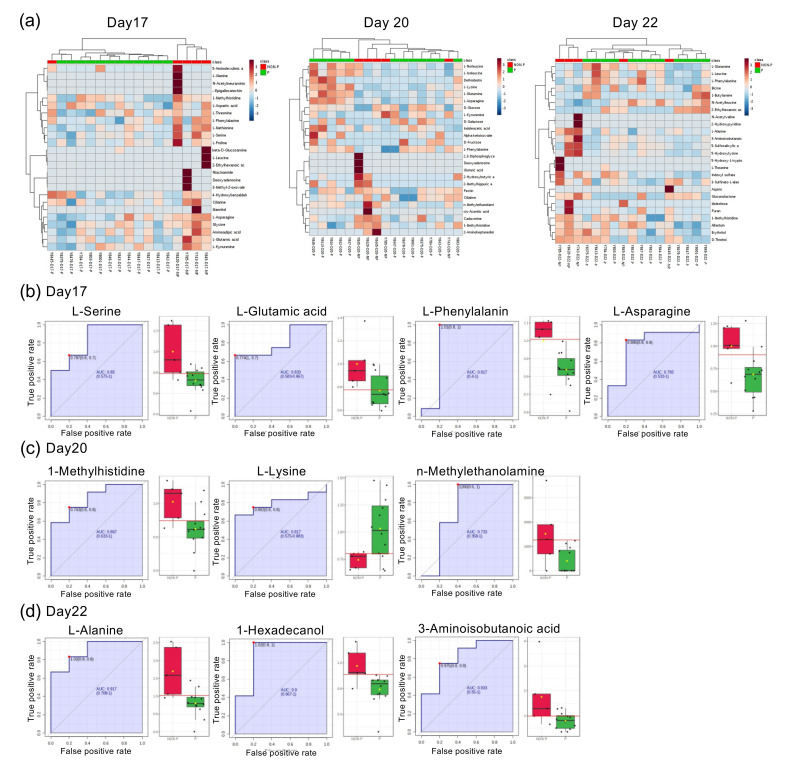
Metabolomic analysis of bovine peripheral blood during the peri-implantation period. (**a**) Heatmap analysis of serum metabolites from serum at days 17, 20, and 22. High-concentration metabolites are shown in red and low-concentration ones are shown in blue. P: pregnant heifers (*n* = 12), NON-P: NP heifers (*n* = 5). (**b**–**d**) Receiver operating characteristic curve (ROC) analysis was performed to assess the predictive power of variables and to measure the optimum cutoff point for NP heifers in days 17 (**b**), 20 (**c**), and 22 (**d**) serum. (**b**–**d**) Box plot next to ROC analysis shows individual sample values from pregnant (P; green) and NP (NON-P; red) heifers.

To study metabolomic profiles between pregnant and NP heifers during peri-implantation periods, serum from peripheral blood at days 0, 7, 14, and 17 (*n* = 3) cyclic, or days 17 (pre-attachment), 20 (during attachment), and 22 (post-attachment) (pregnant (*n* = 12) and NP (*n* = 5)) heifers, were subjected to metabolome analysis. These data were subjected to principal component analysis (PCA), and serum components were divided as either serum from heifers with or without ET, or heifers pregnant or NP (data not shown). Heatmap with serum on days 17, 20, and 22 was also partly divided into pregnant and NP heifers (Figure 1a). We further investigated each metabolic data on days 17, 20, or 22 (Figure 1b), of which serum metabolomic components were partly divided into pregnant or NP groups. In receiver operating characteristic curve (ROC) analysis with day-17 serum metabolomic data, changes in L-serine (area under the curve (AUC) 0.85), L-glutamic acid (AUC 0.83), L-phenylalanine (AUC 0.82), and L-asparagine (AUC 0.78) were significant between pregnant and NP heifers (*p* < 0.05) (Figure 1b). In day 20 ROC analysis, 1-methylhistidine (AUC 0.87), l-lysine (AUC 0.82), and n-methylethanolamine (AUC 0.73) were detected (*p* < 0.05) (Figure 1c). Furthermore, ROC analysis with day-22 serum metabolomic data identified (*p* < 0.05) L-alanine (AUC 0.92), 1-hexadecanol (AUC 0.9), and 2-aminoisobutanoic acid (AUC 0.83) as the amino acid contents that differed between pregnant and NP heifers (Figure 1d).

### 2.2. Comparison of Serum Composition between Pregnant, NP Heifers, and Those with the Estrous Cycle

**Figure 2:**
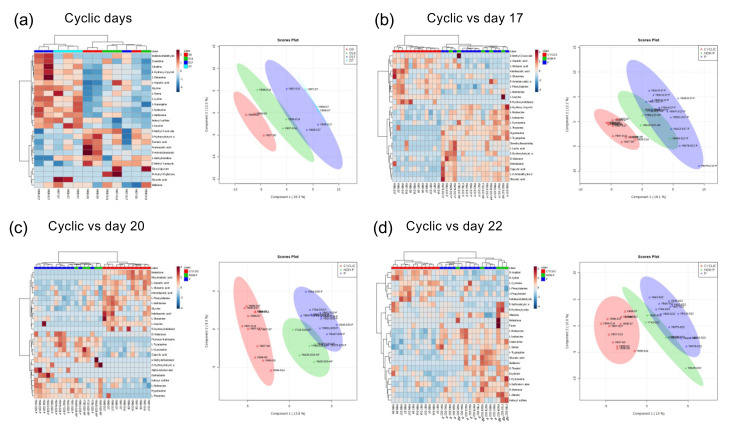
Serum metabolites in cyclic heifers and comparison of those with pregnant and NP heifers. (**a**) Heatmap analysis of serum metabolites in days 0, 7, 14, and 17 (*n* = 3 each day) estrous cycles. Biplot of serum metabolomic components in cyclic heifers (days 0, 7, 14, and 17), produced by the principal component analysis (PCA). (**b**–**d**) Heatmap analysis and PCA of serum metabolites in cyclic heifers and those in day 17 (**b**), 20 (**c**), or 22 (**d**) pregnant and NP heifers. CYCLIC: serum samples from heifers with estrous cycle (red), P: serum from pregnant heifers (green), NON-P: serum from NP heifers (blue).

We next compared the serum metabolomic data from ET heifers (*n* = 17) with those from the estrous cycle (*n* = 3). First, we compared serum metabolites among cyclic days 0, 7, 14, and 17. The heatmap and PCA showed that the estrous cycle data were divided into different cyclic days (Figure 2a). In the serum metabolite comparison of day-17 pregnant and NP heifers with those of cyclic ones, metabolites on cyclic days were separated from those of day-17 pregnant or NP heifers (Figure 2b). However, metabolites from day-17 pregnant heifers were not divided from those of NP heifers. On the day-20 comparison, metabolites of cyclic days were completely separated from those of day-20 pregnant or NP heifers (Figure 2c). Metabolites on day 20 could be further divided into pregnant and NP groups. Similar to D20, metabolites on cyclic days were separated from those of day-22 pregnant and NP heifers (Figure 2d). Metabolites on day 22 could also be divided into pregnant and NP heifers.

### 2.3. Global Proteome Analysis of Serum from Day 20 Pregnant and NP Heifers

**Figure 3:**
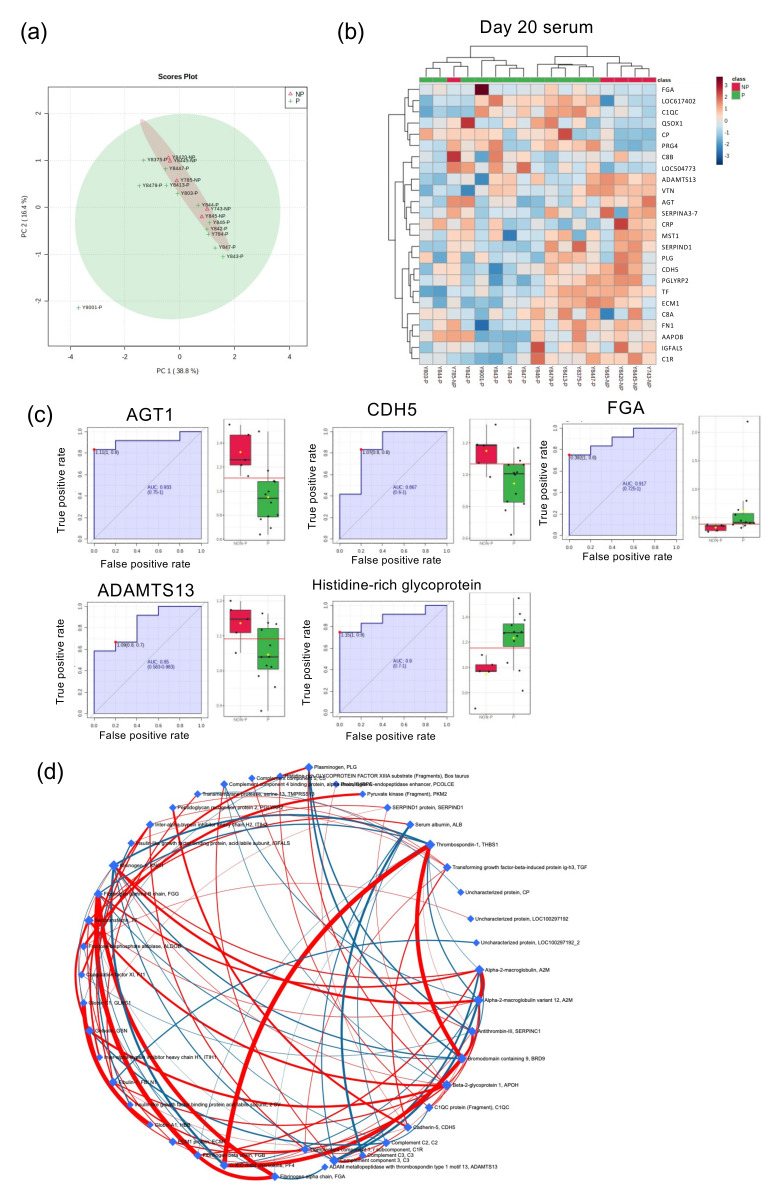
Global proteome analysis of day-20 peripheral blood. (**a**) Biplot produced by PCA of proteins identified by iTRAQ proteome analysis in the serum from day-20 pregnant and NP heifers. (**b**) Heatmap analysis of serum proteins in day-20 pregnant and NP heifers. High-concentration proteins are shown in red and low-concentration proteins are shown in blue. P: serum from pregnant heifers (*n* = 12), NP: serum from NP heifers (*n* = 5). (**c**) ROC analysis was performed to assess the predictive power of serum variables and to measure the optimum cutoff point in day-20 NP heifers. Box plot shows individual sample values from pregnant (green) and NP (red) heifers. (**d**) Network analysis with proteins identified in day-20 serum by iTRAQ. Red or blue lines indicate positive or negative regulation, respectively.

In addition to the metabolomic profiles, we investigated the proteome profile of serum from day-20 pregnant and NP heifers. PCA did not divide protein components in day-20 pregnant heifers from those of NP ones (Figure 3a). Heatmap with top 25 proteins separated protein components in day-20 NP heifers except for one sample (Figure 3b), from which ROC analysis identified five candidate proteins signifying pregnancy failure (*p* < 0.05): angiotensinogen (AGT; AUC 0.93), cadherin-5 (CDH5; AUC 0.87), fibronectin alpha chain (FGA; AUC 0.92), ADAM metallopeptidase with thrombospondin type 1 motif 13 (ADAMTS13; AUC 0.85), and histidine-rich glycoprotein (AUC 0.9) (Figure 3c). Furthermore, the network analysis with all protein profile identified impacting proteins: FGA, FGB, FGG, thrombospondin-1 (THBS1), bromodomain containing 9 (BRD9), and C-X-C motif chemokine ligand 4 (PF4) (Figure 3d).

### 2.4. Identification of Specific Proteins in Day-22 Blood Serum Detecting NP Heifers

**Figure 4:**
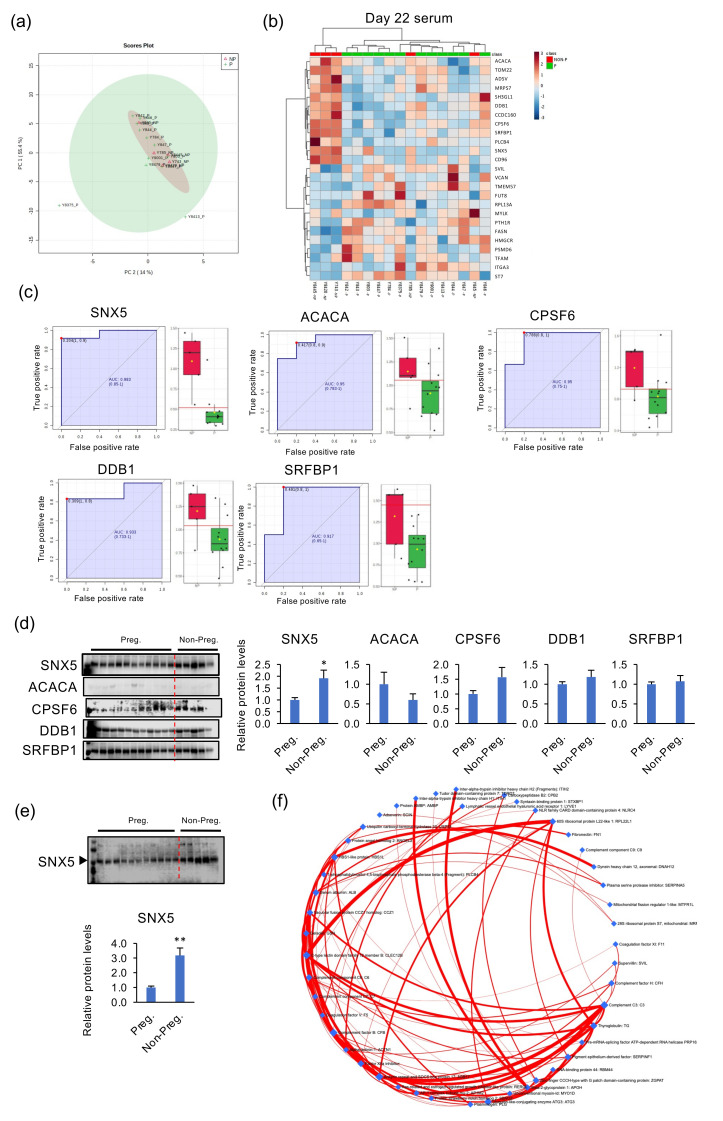
Identification of candidate proteins in day-22 peripheral blood to identify NP heifers from pregnant ones. (**a**) Biplot produced by PCA of proteins identified by iTRAQ proteome analysis in the serum of day-22 pregnant and NP heifers. (**b**) Heatmap analysis of serum proteins in day-22 pregnant and NP heifers. High-concentration proteins are shown in red and low-concentration proteins are shown in blue. P: serum from pregnant heifers (*n* = 12), NP: serum from NP heifers (*n* = 5). (**c**) ROC analysis was performed to assess the predictive power of variables and to measure the optimum cutoff point in the serum from day-22 NP heifers from pregnant ones. Box plot shows individual sample values from pregnant (green) and NP (red) heifers. (**d**) The serum from day-22 pregnant and NP heifers was subjected to western blotting, which revealed the presence of proteins identified by ROC analysis: SNX5, ACACA, CPSF6, DDB1, and SRFBP1. The bar graphs on the right show the relative protein levels. * *p* < 0.05 vs. Preg. (**e**) Western blotting was again conducted only for SNX5. Relative protein levels are shown in the bar graph below. Note that we had 12 pregnant heifers, from which 11 were subjected to the western blotting analysis due to the sample number limitation in our western blot system. ** *p* < 0.01 vs. Preg. (**f**) Network analysis with identified protein data on day 22 by iTRAQ. Red line indicates positive regulation.

We next investigated the proteome profile of serum from day-22 pregnant or NP heifers. Like day-20 serum, PCA did not divide protein components on day-22 pregnant from those of NP heifers (Figure 4a). Heatmap analysis with top 25 proteins separated serum protein components in NP heifers from those of day-22 samples (Figure 4b), of which ROC analysis identified five candidate proteins detecting NP heifers (*p* < 0.05): sorting nexin 5 (SNX5; AUC 0.98), acetyl-CoA carboxylase alpha (ACACA; AUC 0.95), cleavage and polyadenylation specific factor 6 (CPSF6; AUC 0.95), damage specific DNA binding protein 1 (DDB1; AUC 0.93), and serum response factor binding protein 1 (SRFBP1) (Figure 4c). To examine whether these five proteins could be used as markers to detect NP heifers, day-22 peripheral serum samples were subjected to western blotting analysis. SNX5 in the serum from NP heifers was somewhat higher than that in the pregnant heifers (Figure 4d). However, ACACA, CPSF6, DDB1, and SRFBP1 in the serum did not differ between day-22 pregnant and NP heifers. Western blots were again executed using another antibody specific for SNX5, resulting that SNX5 in the NP heifers was higher than that of pregnant heifers on day 22 (Figure 4e). Moreover, the network analysis with day-22 protein profiles identified impacting proteins: component C3 (C3), thyroglobulin (TG), c-type lectin domain family 12 member B (CLEC12B), ankyrin repeat and SOCS box protein 17 (ASB17), and beta-2-glycoprotein1 (APOH) (Figure 4f).

## 3. Discussion

Using PCA and the heatmap with top 25 factors from the metabolome analysis, serum components were divided by pregnant at days 17, 20, and 22 from those of NP heifers. On each pregnant day sample, serum components were partly divided into pregnant and NP groups, some of which were higher in NP heifers than those of pregnant ones. Notably, serum metabolomic components from cyclic days 0, 7, 14, and 17 were distinctive due possibly to circulating P4, which also differed from those of days-17, -20, or -22 NP heifers. These data indicate that serum metabolomic components in NP heifers somewhat differ from those of cyclic ones, but those components were not sufficient enough to separate NP heifers from the pregnant ones during the periods being examined. In the proteome iTRAQ analysis, the heatmap with top 25 proteins separated into pregnant and NP heifers on day 20 or 22. Furthermore, ROC analysis identified five candidate proteins detecting heifers as not pregnant, of which serum SNX5 on day 22 had the highest AUC of 0.983. Using western blotting with day-22 serum, we detected more SNX5 in NP heifers than pregnant ones. These results indicate that peripheral blood on day 22, around two to three days after conceptus attachment to the maternal endometrium is initiated, reflects some differences between pregnant and NP heifers during the early pregnancy period, and suggests that high levels of SNX5 in peripheral blood on day 22 could predict pregnancy failure or those undergoing embryonic losses in ET heifers.

SNX5 encodes a member of the sorting nexin family. Members of this family contain a phox (PX) domain, a phosphoinositide binding domain, and are involved in intracellular trafficking. SNX5 protein functions in endosomal sorting, the phosphoinositide signaling pathway, macropinocytosis, and micropinocytosis [14,15,16]. Macropinocytosis and micropinocytosis are considered essential for providing nutrients from a mother to the fetus. In humans, the trophoblast cells possess various transporters for glucose, amino acids, and fatty acid and are believed to preferentially utilize these low molecular-weight nutrients. However, maternal exposure to nutrient insufficiency will cause restricted nutrient supply to the placenta. The macropinocytosis is adapted as an alternative means of nutrient source to allow sustained fetal growth, which is greatly enhanced during amino acid shortage [17,18]. In addition, it has been reported that SNX5 stimulates autophagy during viral infection [19]. In cattle, amino acids are essential for the survival and development of embryo during early pregnancy periods [20,21]. Moreover, amino acid concentrations in uterine fluids during early pregnancy differ in fertile and subfertile dairy cows [22]. In this study, the metabolome analysis showed that the serum levels of several amino acids differed between pregnant and NP heifers. These observations suggest that the increase in amino acid usages for trophoblast/fetal and placental growth recruits more plasma amino acids to the uterus in pregnant animals, but in the NP heifers, which experienced early embryonic loss, these recruitments do not occur, resulting in transient increase in some amino acids and SNX5 in the plasma.

For the last decade and particularly recently, substantial data on pregnancy detection in cattle have become available: ISGs expression in PBMCs and PMNs [8], CL examination by Doppler ultrasonography [10], and microRNA in extracellular vesicles [12]. In addition, several studies have recently been attempting to identify specific markers for discriminating success or failure of pregnancy in ruminants [22,23,24,25,26,27,28]. However, these methods are not yet applicable in the field. On the other hand, pregnancy-associated glycoproteins (PAGs), produced by mono-, bi- or multi-nucleated trophectodermal cells, are highly released into maternal circulations, and its ELISA detection system is commercially available for domestic ruminants [29]. This detection method enables heifers/cows to identify pregnant animals from those that are not pregnant on day 28 or later. PAGs are pepsin-like aspartic proteinases expressed by mononuclear trophoblast and by binuclear trophoblast cells in cattle and sheep [30,31,32], although PAG function as active proteases is unclear. PAGs are expressed after conceptus implantation and expression increases throughout pregnancy with peak blood concentrations at or near parturition [31]. This study identified SNX5 as a candidate protein for the discrimination of NP heifers from pregnant ones on day 22, and suggests that SNX5 could be usable as a pregnancy test prior to the ELISA test with PAG antibodies. Because these data were based on the use of 20 heifers, further investigation is required to find if SNX5 could become a unique serum protein for reliable pregnancy diagnosis.

In conclusion, this study shows that metabolomic analysis of peripheral blood on days 17, 20, and 22 partly separates pregnant and NP heifers. The proteome iTRAQ analysis also separated serum protein components between pregnant and NP heifers on day 20 or 22. Furthermore, ROC analysis identified five candidate proteins detecting pregnancy failure, of which SNX5 was detected as a potential target for discrimination between NP heifers and pregnant ones. These results indicate that peripheral blood on day 22 can be used for a pregnancy test, and suggest that high levels of serum SNX5 on day 22 could predict females undergoing embryonic loss in heifers.

## 4. Materials and Methods

### 4.1. Collection of Bovine Blood Samples

All animal procedures in this study were performed in accordance with the guidelines of the Committee for Experimental Animals at Zen-noh Embryo Transfer Center (Hokkaido, Japan), with the approval of the Institutional Animal Care and Use Committee of Zen-noh Embryo Transfer Center (Approval number: ZET20190628). All animals used were raised and kept at this center throughout the course of this experimentation. This study was carried out in compliance with the ARRIVE guidelines. Estrous synchronization, superovulation, and embryo transfer (ET) processes were performed as previously described [33]. Holstein heifers (14~16 months old, *n* = 24), exhibiting regular estrous cycles, were given a single injection of 0.75 mg prostaglandin F2α (d-cloprostenol (Dalmazin), Kyoritu Pharmaceutical Co., Tokyo, Japan) to synchronize their estrous cycles and the heifers exhibited behavioral estrus 40–48 h after the injection (*n* = 20, day 0 = day of estrus). Twenty heifers were then divided into two groups, ET (*n* = 17) and non-ET (*n* = 3). For ET processes, in vivo-fertilized day-7 embryos were collected from super-ovulated and artificially inseminated (AI) Japanese black cows (3~5 years old, *n* = 5). Single blastocyst derived from the superovulation/AI procedure was then transferred non-surgically into the uterine horn of Holstein heifers, ipsilateral to the corpus luteum, on day 7 of the estrous cycle [33].

Blood samples were collected from ET heifers on pregnancy days 17, 20, and 22. In addition, blood samples from non-ET heifers (*n* = 3) were collected on days 0, 7, 14, and 17 of the estrous cycle. The diagnosis of pregnancy or non-pregnancy (NP), indicative of early embryonic loss, was conducted by real-time B-mode ultrasonography (Convex scanner HS-1500, Honda electronics Co., Ltd., Toyohashi, Japan) on day 30 of gestation, from which blood samples were divided into pregnant or NP groups. After sorting of blood samples, albumin was removed from serum samples by ProMax Albumin Removal Kit (Polysciences, Warrington, PA, USA).

### 4.2. Metabolome Analysis

A serum metabolomics analysis was performed using GC/MS as described previously [34] with some modifications. In brief, a sample of 50 μL of serum was mixed with 5 μL of 1 mg/mL 2-isopropylmalic acid (Sigma-Aldrich, St. Louis, MO, USA) in distilled water as an internal standard, and 250 μL of methanol–chloroform–water (2.5:1:1) mixture. Then samples were lyophilized, and added with 40 μL of 20 mg/mL methoxyamine hydrochloride (Sigma-Aldrich), dissolved in pyridine for oximation. After mixing, the samples were shaken for 90 min at 30 °C. Next, 20 μL of N-methyl N-trimethylsilyl-trifluoroacetamide (GL Science, Tokyo, Japan) was added for trimethylsilylation, and the mixture was incubated at 37 °C for 45 min. The sample was subjected to GC/MS (GCMS QP2010-Ultra; Shimadzu, Kyoto, Japan). The Shimadzu Smart Metabolites Database (Shimadzu) was used to identify metabolites. Samples were normalized by a pooled all sample. All data are presented in Supplementary Tables S1 and S2 [35]. A metabolic pathway analysis was performed using MetaboAnalyst [36]. Metabolites that significantly differed between two groups were subjected to an enrichment analysis (http://www.metaboanalyst.ca/faces/upload/EnrichUploadView.xhtml, accessed on 1 June 2021).

### 4.3. iTRAQ Analysis

A global analysis of proteins using iTRAQ analysis was performed as described previously [37]. Briefly, serum samples from ET heifers on days 17, 20 or 22 and those without ET were resuspended in 30 µL iTRAQ lysis buffer (50 mM TAEB, 0.1% SDS). Total protein (100 µg) was subjected to trypsin digestion and then reacted with appropriate iTRAQ reagent according to the manufacturer’s instructions. Sample fractionation was performed with an Agilent 3100 OFFGEL Fractionator (Agilent Technologies, Santa Clara, CA, USA). Furthermore, mass spectrometry analysis was performed with a Thermo Scientific LTQ Orbitrap XL mass spectrometer (Thermo Fisher Scientific, Waltham, MA, USA). Mascot software was used to simultaneously identify and quantify proteins and those are presented in Supplementary Tables S3 and S4 [35]. PCA, ROC, and network analyses were performed using MetaboAnalyst.

### 4.4. Western Blot Analysis

Serum samples were separated through SDS-PAGE and were then transferred onto polyvinylidene difluoride (PVDF) membranes (Bio-Rad, Hercules, CA, USA). After blocking with Block Ace reagent (DS Pharma Biomedical, Osaka, Japan), membranes were incubated with goat polyclonal anti-SNX5 (1:2000, ab5983, abcam, Tokyo, Japan), rabbit polyclonal anti-SRFBP1 (1:2000, ab109598, abcam), rabbit polyclonal anti-DDB1 (1:2000, ab97522, abcam), rabbit monoclonal anti-CPSF6 (1:2000, ab175237, abcam), or mouse monoclonal anti-ACACA (1:2000, ab205883, abcam) antibody. The second western blot was conducted with rabbit polyclonal anti-SNX5 (1:2000, SAB2102260, Sigma-Aldrich, Tokyo, Japan) antibody. Immunoreactive bands were detected using enhanced chemiluminescence (EMD Millipore, Temecula, CA, USA) after incubation with horseradish peroxidase labeled anti-mouse, rabbit, or goat IgG (1:5000, Vector Laboratories, Burlingame, CA, USA). Signals were detected using C-DiGit Blot Scanner (LI-COR) and then band density was assessed with Image Studio DiGit software (version 5.2) [38]. The whole blot can be found at supplementary materials (Figures S2 and S3).

### 4.5. Statistical Analysis

All experimental data represent the results obtained from three or more independent experiments each with triplicate assays. Data were expressed as the mean ± SEM. A *p*-value < 0.05 was considered statistically significant. The *p*-value was used to evaluate the significance of the ROC curve or other analyses in this study.

## Data Availability

All metabolome and proteome data were deposited in the Figshare (https://doi.org/10.6084/m9.figshare.16778674.v1).

## Supplementary Materials

The following supporting information can be downloaded at: https://www.mdpi.com/article/10.3390/life12020309/s1, Figure S1: Flow-chart of sample collection; Figure S2. The serum from day-22 pregnant and NP heifers was subjected to western blotting, which revealed the presence of proteins identified by ROC analysis: SNX5, ACACA, CPSF6, DDB1, and SRFBP1. The bar graphs on the right show the relative protein levels; Figure S3. Western blotting was again conducted only for SNX5. Relative protein levels are shown in the bar graph below. Note that we had 12 pregnant heifers, from which 11 were subjected to the western blotting analysis due to the sample number limitation in our western blot system.

## References

[1] Roberts R.M., Cross J.C., Leaman D.W. (1992). Interferons as hormones of pregnancy. Endocr. Rev..

[2] Spencer T.E., Becker W.C., George P., Mirando M.A., Ogle T.F., Bazer F.W. (1995). Ovine interferon-tau regulates expression of endometrial receptors for estrogen and oxytocin but not progesterone. Biol. Reprod..

[3] Spencer T.E., Bazer F.W. (1996). Ovine interferon tau suppresses transcription of the estrogen receptor and oxytocin receptor genes in the ovine endometrium. Endocrinology.

[4] Forde N., Lonergan P. (2017). Interferon-tau and fertility in ruminants. Reproduction.

[5] Sponchiado M., Gomes N.S., Fontes P.K., Martins T., Del Collado M., Pastore A.A., Pugliesi G., Nogueira M.F.G., Binelli M. (2017). Pre-hatching embryo-dependent and -independent programming of endometrial function in cattle. PLoS ONE.

[6] Bridi A., Bertolin K., Rissi V.B., Mujica L.K.S., Glanzner W.G., de Macedo M.P., Comim F.V., Gonçalves P.B.D., Antoniazzi A.Q. (2018). Parthenogenetic bovine embryos secrete type I interferon capable of stimulating ISG15 in luteal cell culture. Anim. Reprod..

[7] Ruhmann B., Giller K., Hankele A.K., Ulbrich S.E., Schmicke M. (2017). Interferon-τ induced gene expression in bovine hepatocytes during early pregnancy. Theriogenology.

[8] Kizaki K., Shichijo-Kizaki A., Furusawa T., Takahashi T., Hosoe M., Hashizume K. (2013). Differential neutrophil gene expression in early bovine pregnancy. Reprod. Biol. Endocrinol..

[9] Soumya N.P., Das D.N., Jeyakumar S., Mondal S., Mor A., Mundhe U.T. (2017). Differential expression of ISG 15 mRNA in peripheral blood mononuclear cells of nulliparous and multiparous pregnant versus non-pregnant Bos indicus cattle. Reprod. Domest. Anim. Zuchthyg..

[10] Pugliesi G., Miagawa B.T., Paiva Y.N., França M.R., Silva L.A., Binelli M. (2014). Conceptus-induced changes in the gene expression of blood immune cells and the ultrasound-accessed luteal function in beef cattle: How early can we detect pregnancy?. Biol. Reprod..

[11] Hansen T.R., Sinedino L.D.P., Spencer T.E. (2017). Paracrine and endocrine actions of interferon tau (IFNT). Reproduction.

[12] Rocha C.C., da Silva Andrade S.C., de Melo G.D., Motta I.G., Coutinho L.L., Gonella-Diaza A.M., Binelli M., Pugliesi G. (2020). Early pregnancy-induced transcripts in peripheral blood immune cells in Bos indicus heifers. Sci. Rep..

[13] Bazer F.W., Spencer T.E., Johnson G.A., Burghardt R.C., Wu G. (2009). Comparative aspects of implantation. Reproduction.

[14] Trousdale C., Kim K. (2015). Retromer: Structure, function, and roles in mammalian disease. Eur. J. Cell Biol..

[15] Bonifacino J.S., Hurley J.H. (2008). Retromer. Curr. Opin. Cell Biol..

[16] Itai N., Shimazu T., Kimura T., Ibe I., Yamashita R., Kaburagi Y., Dohi T., Tonozuka T., Takao T., Nishikawa A. (2018). The phosphorylation of sorting nexin 5 at serine 226 regulates retrograde transport and macropinocytosis. PLoS ONE.

[17] Shao X., Cao G., Chen D., Liu J., Yu B., Liu M., Li Y.X., Cao B., Sadovsky Y., Wang Y.L. (2021). Placental trophoblast syncytialization potentiates macropinocytosis via mTOR signaling to adapt to reduced amino acid supply. Proc. Natl. Acad. Sci. USA.

[18] Palm W., Park Y., Wright K., Pavlova N.N., Tuveson D.A., Thompson C.B. (2015). The Utilization of Extracellular Proteins as Nutrients Is Suppressed by mTORC1. Cell.

[19] Dong X., Yang Y., Zou Z., Zhao Y., Ci B., Zhong L., Bhave M., Wang L., Kuo Y.C., Zang X. (2021). Sorting nexin 5 mediates virus-induced autophagy and immunity. Nature.

[20] Forde N., Simintiras C.A., Sturmey R., Mamo S., Kelly A.K., Spencer T.E., Bazer F.W., Lonergan P. (2014). Amino acids in the uterine luminal fluid reflects the temporal changes in transporter expression in the endometrium and conceptus during early pregnancy in cattle. PLoS ONE.

[21] Gilbreath K.R., Bazer F.W., Satterfield M.C., Wu G. (2021). Amino Acid Nutrition and Reproductive Performance in Ruminants. Adv Exp. Med. Biol..

[22] Meier S., Mitchell M.D., Walker C.G., Roche J.R., Verkerk G.A. (2014). Amino acid concentrations in uterine fluid during early pregnancy differ in fertile and subfertile dairy cow strains. J. Dairy Sci..

[23] Pohler K.G., Reese S.T., Franco G.A., Oliveira R.V., Paiva R., Fernandez L., de Melo G., Vasconcelos J.L.M., Cooke R., Poole R.K. (2020). New approaches to diagnose and target reproductive failure in cattle. Anim. Reprod..

[24] Ott T.L. (2020). Immunological detection of pregnancy: Evidence for systemic immune modulation during early pregnancy in ruminants. Theriogenology.

[25] Ott T.L. (2019). Symposium review: Immunological detection of the bovine conceptus during early pregnancy. J. Dairy Sci..

[26] Kunii H., Kubo T., Asaoka N., Balboula A.Z., Hamaguchi Y., Shimasaki T., Bai H., Kawahara M., Kobayashi H., Ogawa H. (2021). Loop-mediated isothermal amplification (LAMP) and machine learning application for early pregnancy detection using bovine vaginal mucosal membrane. Biochem. Biophys. Res. Commun..

[27] Kunii H., Koyama K., Ito T., Suzuki T., Balboula A.Z., Shirozu T., Bai H., Nagano M., Kawahara M., Takahashi M. (2018). Hot topic: Pregnancy-induced expression of interferon-stimulated genes in the cervical and vaginal mucosal membranes. J. Dairy Sci..

[28] Green J.C., Okamura C.S., Poock S.E., Lucy M.C. (2010). Measurement of interferon-tau (IFN-tau) stimulated gene expression in blood leukocytes for pregnancy diagnosis within 18-20d after insemination in dairy cattle. Anim. Reprod. Sci..

[29] Green J.A., Parks T.E., Avalle M.P., Telugu B.P., McLain A.L., Peterson A.J., McMillan W., Mathialagan N., Hook R.R., Xie S. (2005). The establishment of an ELISA for the detection of pregnancy-associated glycoproteins (PAGs) in the serum of pregnant cows and heifers. Theriogenology.

[30] Wooding F., Roberts R., Green J. (2005). Light and electron microscope immunocytochemical studies of the distribution of pregnancy associated glycoproteins (PAGs) throughout pregnancy in the cow: Possible functional implications. Placenta.

[31] Wallace R.M., Pohler K.G., Smith M.F., Green J.A. (2015). Placental PAGs: Gene origins, expression patterns, and use as markers of pregnancy. Reproduction.

[32] Green J.A., Xie S., Roberts R.M. (1998). Pepsin-related molecules secreted by trophoblast. Rev. Reprod..

[33] Ideta A., Urakawa M., Aoyagi Y., Saeki K. (2007). Early development in utero of bovine nuclear transfer embryos using early G1 and G0 phase cells. Cloning Stem Cells.

[34] Pu S., Usuda K., Nagaoka K., Watanabe G. (2019). Heat challenge influences serum metabolites concentrations and liver lipid metabolism in Japanese quail (Coturnix japonica). J. Vet. Med. Sci..

[35] Kusama K.I., Kazuhiko I. (2021). Supplementary Tables.xlsx. Figshare. Dataset.

[36] Xia J., Wishart D.S. (2011). Web-based inference of biological patterns, functions and pathways from metabolomic data using MetaboAnalyst. Nat. Protoc..

[37] Kusama K., Bai R., Ideta A., Aoyagi Y., Okuda K., Imakawa K. (2016). Regulation of epithelial to mesenchymal transition in bovine conceptuses through the interaction between follistatin and activin A. Mol. Cell. Endocrinol..

[38] Kusama K., Bai R., Imakawa K. (2018). Regulation of human trophoblast cell syncytialization by transcription factors STAT5B and NR4A3. J. Cell. Biochem..

