# Supplementary material for: Characterization of Serum Metabolome and Proteome Profiles Identifies SNX5 Specific for Pregnancy Failure in Holstein Heifers
Source: Life (Basel). 2022 Feb 18;12(2):309. doi: 10.3390/life12020309 (PMC8877674; doi:10.3390/life12020309)
Supplement: Supplementary file 1 [file life-12-00309-s001.zip › life-1565381-supplementary.pdf]

life

Article

Characterization of Serum Metabolome and Proteome Profiles Identifies SNX5 Specific for Pregnancy Failure in Holstein Heifers

Kazuya Kusama [1], Rulan Bai [2], Yuta Matsuno [3], Atsushi Ideta [4], Toshihiro Sakurai [5], Kentaro Nagaoka [6], Masatoshi Hori [7] and Kazuhiko Imakawa [3,*]

Supplementary Materials:

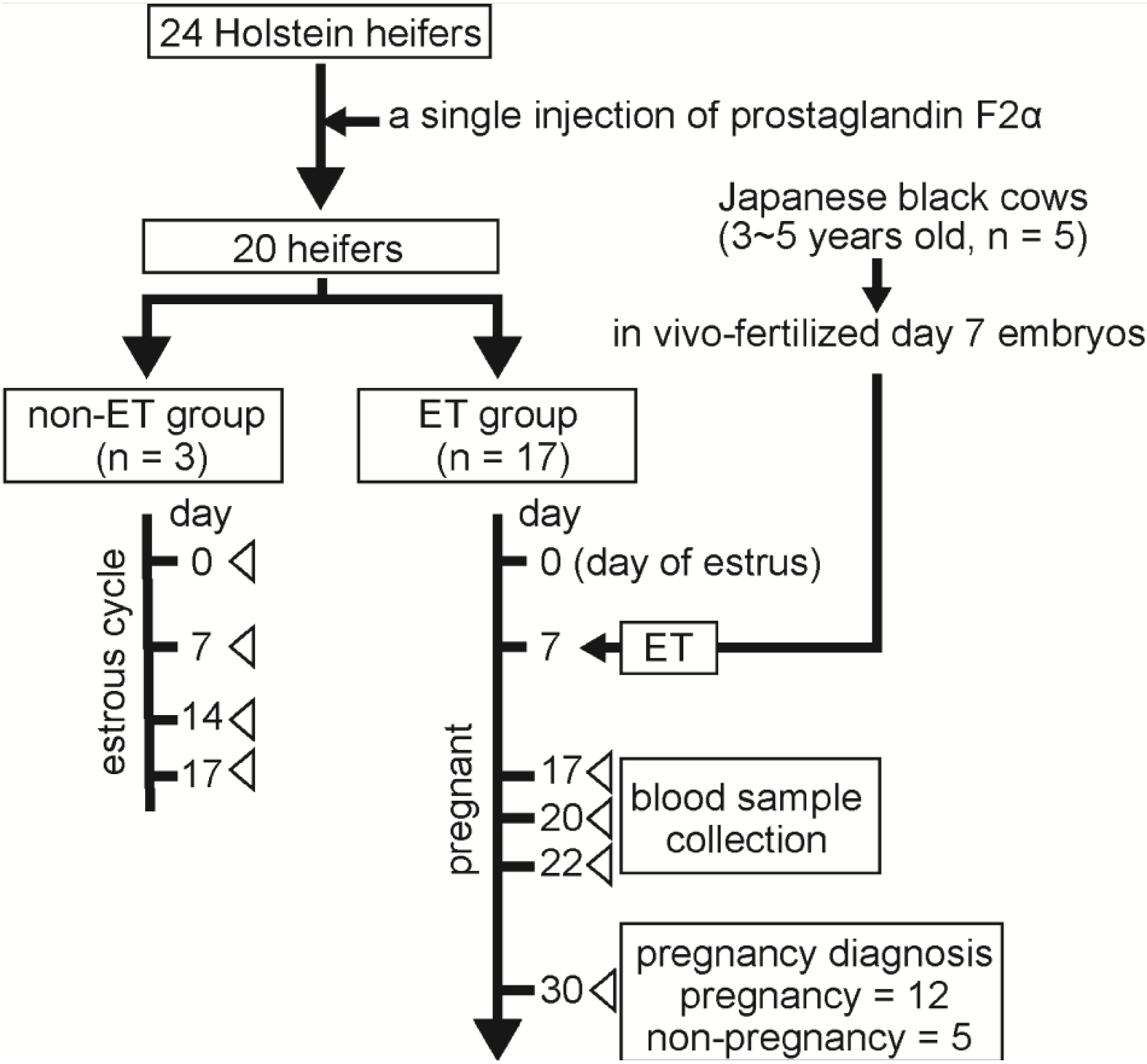

Figure S1. Flow-chart of sample collection.

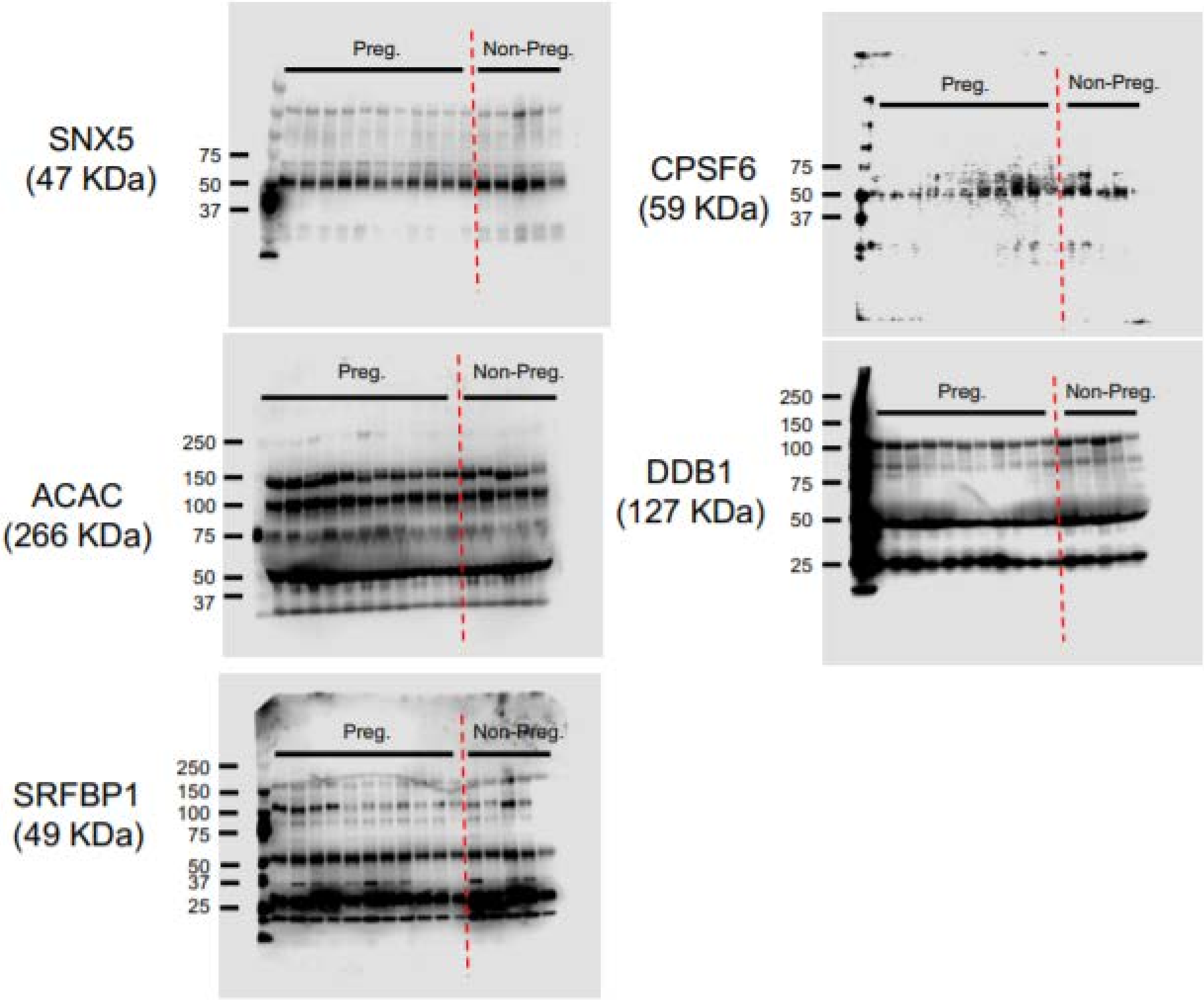

Figure S2. The serum from day-22 pregnant and NP heifers was subjected to western blotting, which revealed the presence of proteins identified by ROC analysis: SNX5, ACACA, CPSF6, DDB1, and SRFBP1. The bar graphs on the right show the relative protein levels.

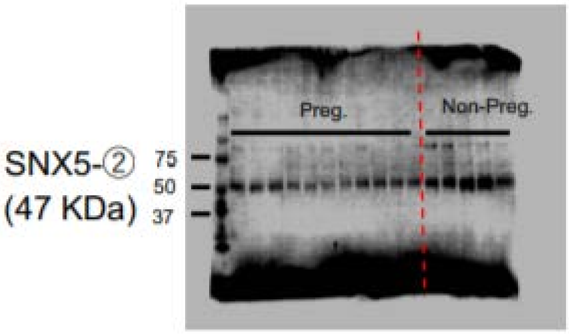

Figure S3. Western blotting was again conducted only for SNX5. Relative protein levels are shown in the bar graph below. Note that we had 12 pregnant heifers, from which 11 were subjected to the western blotting analysis due to the sample number limitation in our western blot system.
